# NPTX1-related oculomotor apraxia: an intra-hemispheric disconnection disorder

**DOI:** 10.1007/s00415-022-11057-3

**Published:** 2022-03-14

**Authors:** Christoph Helmchen, Philipp J. Koch, Gabriel Girard, Norbert Brüggemann, Björn Machner, Andreas Sprenger

**Affiliations:** 1 Department of Neurology, University Hospital Schleswig-Holstein, Campus LübeckRatzeburger Allee 160, 23538 Lübeck, Germany; 2 Center of Brain, Behavior and Metabolism (CBBM), University of Lübeck, Ratzeburger Allee 160, 23562 Lübeck, Germany; 3 CIBM Center for BioMedical Imaging, CH-1015 Lausanne, Switzerland; 4 Radiology Department, Centre Hospitalier Universitaire Vaudois and University of Lausanne, CH-1011 Lausanne, Switzerland; 5 Signal Processing Laboratory (LTS5), School of Engineering, École Polytechnique Fédérale de Lausanne, CH-1015 Lausanne, Switzerland; 6 Institute of Psychology II, University Lübeck, Lübeck, Germany

Dear Sirs,

Oculomotor apraxia (OMA) is a rare and heavily disabling neurological disorder causing severe difficulties in the initiation and maintenance of voluntary eye movements when the head is stationary. If patients try to initiate saccades, they are grossly delayed and hypometric (stair-case). In contrast, patients can initiate large voluntary saccades when gaze is performed with combined eye–head or lid movements [1]. Congenital forms [2], commonly known as infantile-onset saccade initiation delay, preferably affect horizontal eye movements and occur in various genetic disorders, e.g., in oculomotor apraxia type 1 and type 2 [3], and ataxia telangiectasia [4].

Acquired forms of OMA are rare [5] as OMA in patients probably requires bilateral fronto-parietal damage and involvement of fronto-collicular projections to the superior colliculus (SC) [5–7]. In the monkey, lesions of the frontal eye field (FEF) in the frontal cortex (Brodmann area 8) in combination with the posterior eye field [8] or the SC [9] elicit severe loss of voluntary eye movements. Apart from FEF lesions, associated structural brain abnormalities in OMA patients include vermal atrophy/hypoplasia [10] and the agenesis of the corpus callosum [11]. The latter may be a potential structural lesion site to account for the hypothesized inter-hemispheric abnormalities in OMA [11, 12].

The aim of this study was to test competing pathophysiological hypotheses by functional and structural MRI, stating that OMA is related to either abnormal (i) *inter-*hemispheric [11, 12] or (ii) *intra-*hemispheric connectivity between the FEF and related oculomotor structures (oculomotor network) or (iii) both mechanisms. We tested these hypotheses in a patient with an adult-onset progressive OMA and a positive family history, in whom we recently identified a novel mutation in the *Neuronal Pentraxin 1* (*NPTX1*) gene [13], with strong gene expression patterns in the frontal cortex.

The 58-year-old female patient developed visual symptoms at the age of 43 years. Symptoms progressed over 15 years as she developed severe OMA with the inability to initiate horizontal saccades and smooth pursuit eye movements. Similar symptoms were reported by her brother, father and her uncle [13]. The index patient, her brother and father were tested positive for the missense mutation in the *NPTX1* gene. Unfortunately, we could not establish a contact to the patient’s uncle (the father’s brother). On examination under head-fixed conditions, there was severe horizontal OMA but no optic ataxia, neglect, or right–left disorientation and no additional signs of oculomotor cerebellar dysfunction, specifically no gaze-holding deficit (*Suppl. video*). Vestibular responses to caloric irrigation, rotation chair and quantitative head impulse test, cranial and spinal MRI, and nerve conduction studies were normal. Laboratory tests were normal, including CSF, onco-neural and GAD antibodies, alpha feto-protein, cholesterol, albumin. There were neither vascular skin lesions nor pigmentary retinal degeneration. Spinocerebellar ataxias were genetically ruled out. However, a new recurrent missense mutation in the *NPTX1* gene (p.G389R) was identified, as well as in her brother and father [13].

The study was approved by the Ethics Committee of the University of Lübeck (20-208) and performed in accordance with the ethical standards laid down in the 1964 Declaration of Helsinki and its later amendments. The patient gave written informed consent. For comparison, six age-matched (mean age 57.2 years; 4 female) healthy control subjects were examined with the same behavioral and imaging paradigms. Details on the functional and structural imaging methods are listed in the *Supplements*. Apart from the eye movement recordings in the MRI scanner (video-based eyetracker Eyelink 1000Plus, 1000 Hz, SR Research Ltd., Ontario, Canada), we investigated the same paradigms in the lab under head-stationary and head-free conditions in the dark: fixation, reflexive visually guided saccades, self-paced and saccades to memorized (“imagined”) target locations, and smooth pursuit of a slowly sinusoidally moving visual target. As she was only able to perform only a few and very small horizontal saccades, we used 2.5°–7.5° amplitudes in the MRI scanner. Horizontal and vertical eye positions were analyzed offline using Matlab^®^ (R2019b, The Mathworks Inc., Natick, MA, USA). Eye position data were calibrated and filtered (Gaussian filter, 100 Hz). Eye velocity was calculated as the difference of median eye position of four data points before and after the actual data point. Vertical and horizontal smooth pursuit eye movements were initiated by a slowly sinusoidally (horizontally and vertically) moving visual target (± 16° with a frequency of 0.2 Hz; max. velocity = 20°/s).

Behavioral data: Latencies of the patient’s few purely horizontal reflexive saccades were excessively increased (1197.7 ± 300.8 ms; age-matched control subjects: 225.9 ± 28.5 ms), in contrast to her vertical saccades (446.1 ± 112.5 ms; control: 248.1 ± 40 ms). During the head-fixed condition, she could elicit horizontal saccades when combined with a vertical component, resulting in a diagonal trajectory (with shorter latencies 589.9 ± 240.5 ms; controls: 231.2 ± 28 ms) (Fig. 1A). Horizontal (gain 0.51 ± 0.5) more than vertical (gain 0.66 ± 0.2) and oblique (gain 0.72 ± 0.3) saccades were severely hypometric. Self-paced saccades to stationary visual targets or to remembered (imagined) targets from a grid (9 targets each 5° apart that she had explored for 20 s before; [14]) revealed missing or only very small horizontal and larger but still hypometric vertical saccades. Clinically, she was not able to execute anti-saccades.

**Fig. 1:**
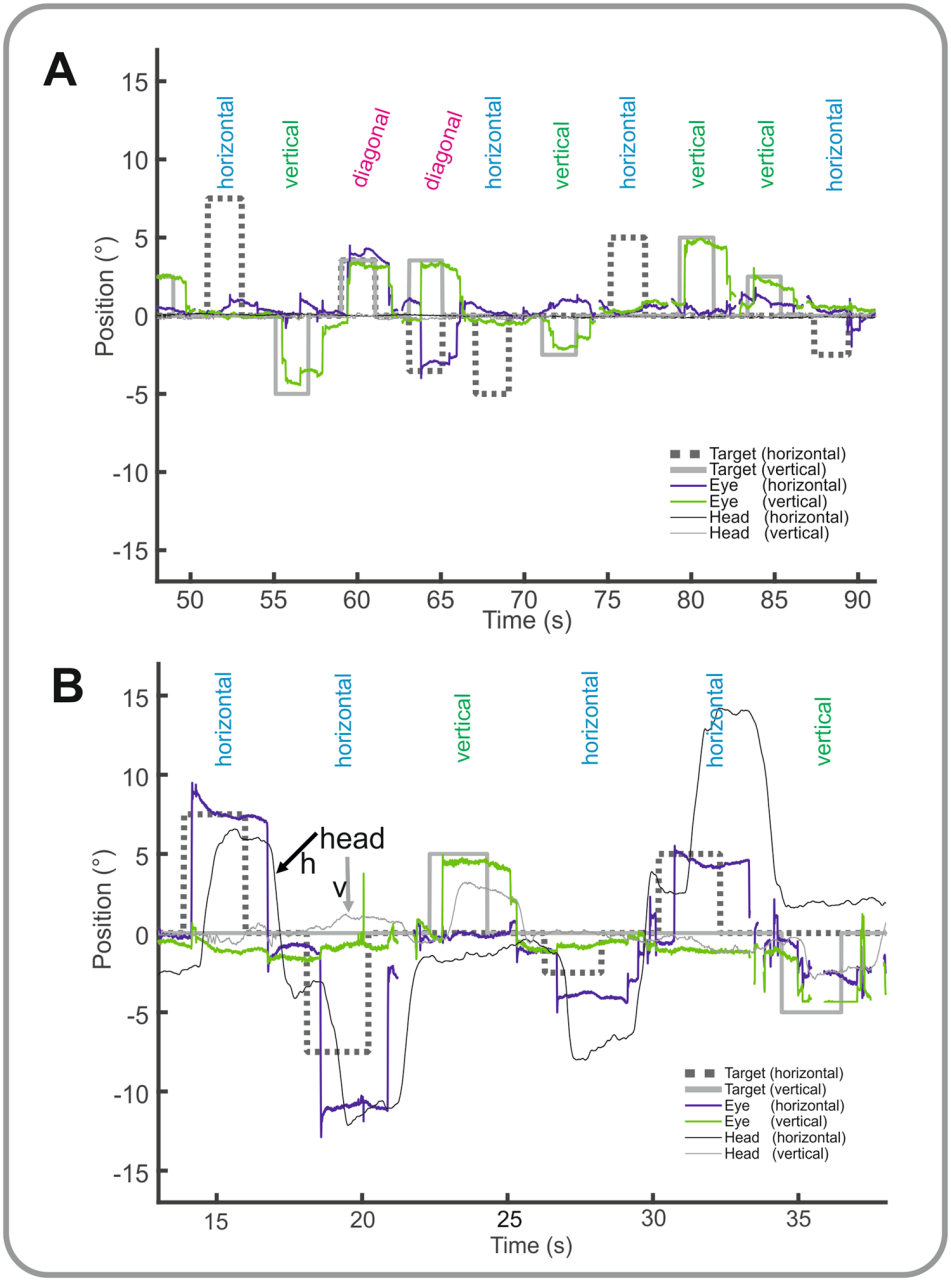
Behavioral data. Horizontal (blue) and vertical (green) visually guided saccades are shown under head-fixed (**A**) and head-free (**B**) conditions. During head-fixed condition (**A**), there are virtually no horizontal visually guided saccades (horizontal target step = dashed line) while she executes vertical saccades during vertical target displacements (gray thick line). During diagonal target displacements, horizontal saccades can be executed with a reasonable amplitude. With the head-free (**B**), pure horizontal saccades can be elicited with nearly normal amplitude when combined with target-directed head movements (thin gray lines indicate vertical and thicker horizontal head movements)

Under head-free conditions, saccades were regularly accompanied by head and blink movements (*Suppl.video*) and their latencies became significantly shorter (481.7 ± 210.7 ms; vertical: 390.9 ± 112.5 ms) (Fig. 1B). Vertical and horizontal saccade velocities were normal (e.g., horizontal 5° = 170°/s; vertical 5° = 150°/s; controls: 180°/s). Horizontal (1.1 ± 0.2) and oblique (1.02 ± 0.4) saccade gain normalized with combined head and blink movements, while vertical gain was still slightly low (0.68 ± 0.2) and oblique (gain 0.72 ± 0.3).

Smooth pursuit: With the head fixed, she was hardly able to perform horizontal smooth pursuit eye movements during a sinusoidally horizontal moving target of (± 6.6° amplitude, 0.3 Hz, peak velocity 10°/sec: velocity gain = 0.22). Vertical smooth pursuit could be performed but was heavily impaired (velocity gain = 0.32). Pursuit performance was much better with the head-free (horizontal velocity gain: 0.58, vertical: 0.35).

There was neither spontaneous eye drift, spontaneous or gaze-evoked nystagmus nor square wave jerks during fixation of a target at gaze straight ahead in the light or darkness.

*Functional imaging (fMRI)*: using a block design, functional activations in the FEF and the supplementary eye field (SEF) mask (Fig. 2C) on both sides were analyzed for the patient and healthy controls separately (Fig. 2A + B, false discovery rate, FDR), for healthy controls: *p* < 0.002 (unc.), > 20 voxel; and *p* < 0.05 corr. for the patient). Group comparison of bilateral FEF (eTable 1*)* activations during visually guided, self-paced and saccades to remembered targets were much weaker in the patient compared to the healthy control subjects (Fig. 2). Case–control studies are unlikely to show statistically significant differences, but deviation (z-value) from the control group mean was below percent range of 5% for the patient in the imagined saccade task in the right FEF (PR = 4.66%), in the visually guided saccade task bilaterally in the SEF (left: PR = 3.59%, right: PR = 1.6%) and in the imagined saccade task in the right SEF (PR = 3.51%).

**Fig. 2:**
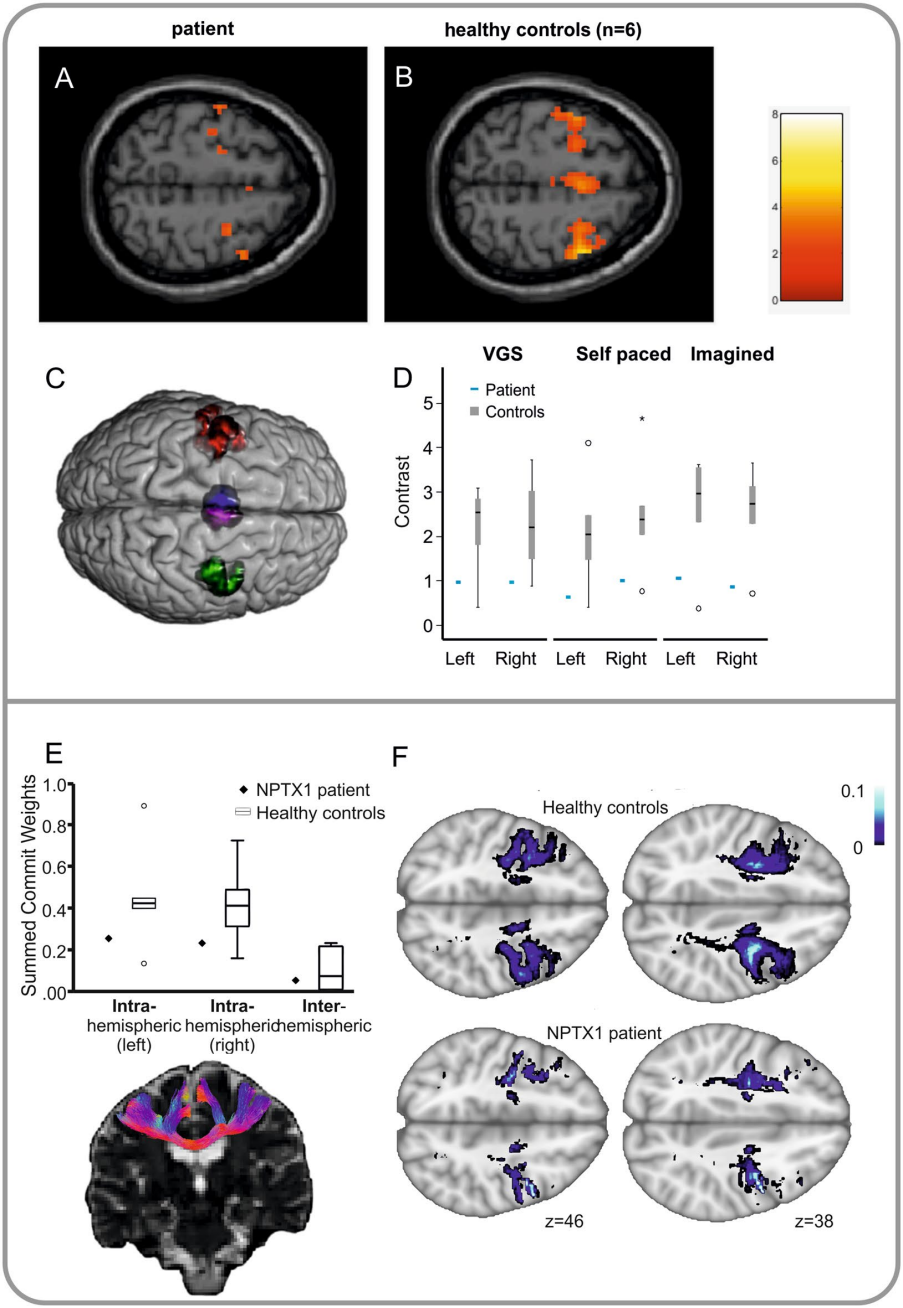
Functional MRI. **A−C**: Neural activity (axial slices) in the mask comprising FEF and SEF (**C**, **eMethods**) during visually guided horizontal and vertical saccades (block design) is shown. FEF and SEF activity during saccades is lower in the patient (**A**) compared to six healthy control subjects (**B**). The group-related contrast difference of brain activity (contrast estimates) in bilateral FEF of the patient (blue bar, **D**) is much lower compared to the healthy control subjects (gray), during different types of saccades (visually guided = VGS, self-paced, i.e., scanning, and memory-guided, i.e., imagined saccades). *Structural connectivity* (diffusion weighted imaging): **E** The mean group structural connectivity, measured by the summed COMMIT weights [16] of the oculomotor network in healthy controls (mean with SD) is shown in box plots and the patient’s value in black squares for *intra*-hemispheric connections of the right and left hemisphere separately and the *inter*-hemispheric connectivity (on the right). Note that the patient’s connectivity is much lower within each hemisphere compared to the control group (white boxes), while there is no difference in the inter-hemispheric connectivity between patient and controls. Below, samples of the streamline estimates for *inter*-hemispheric connections are shown in one representative healthy subject. **F** The density of COMMIT-weighted white matter fascicles of the oculomotor network within each voxel (Tract density imaging, TDI) is projected on MNI standard brain for the patient and the average of the healthy control subjects at representative axial levels (*z* values given)

The structural connectivity of intra-hemispheric connections within the defined oculomotor network [comprising the SEF, the parietal eye field (PEF), the dorsolateral prefrontal cortex (DLPFC), the caudate nucleus, the SC and the pons] from FEF was grossly lower in the patient compared to the healthy control subjects, while there was no group-related difference in the inter-hemispheric connectivity (Fig. 2E, F). The differences for the summed COMMIT Weights in Fig. 2E just failed to become significant with the z-analysis, likely to be due by the small group size.

Our behavioral eye and head movement recordings in this patient with a novel *NPTX1* mutation revealed profound OMA for horizontal voluntary eye movements (saccades and smooth pursuit), in the absence of oculomotor cerebellar signs. Its predominant gene expression pattern is in the cerebellum and the frontal cortex, including the dorsolateral prefrontal cortex (Brodmann Area 9), being involved in the planning, preparation and the executive control of voluntary saccades, and the FEF (Brodmann area 8) [13]. While the FEF is involved in intentional saccades, the SEF controls their preparation and the PEF reflexive saccades. In contrast to another previously described family carrying the G389R loss-of-function mutation [13], our cases did not present with obvious cerebellar signs. NPTX1 shows highest expression levels in the cerebellum followed by cortex, particularly anterior cingulate and prefrontal cortex. This observation highlights that different phenotypes may be associated with pathology in plausible brain regions. The absence of cerebellar signs and atrophy in our family indicates that high expression levels of mutated proteins are not always associated with a phenotype although long-term follow-up is required to assess whether such abnormalities may develop later in the disease course.

The relative ease to overcome the patient’s OMA by blinking and head movements suggests these sites, i.e., FEF and SEF, to be involved in this OMA, since the FEF, but not the posterior parietal cortex, is active during blinking [15]. With the statistical limitations of single-case studies, we provide some evidence for an abnormally reduced structural connectivity within the defined oculomotor network of each hemisphere. In contrast, the indistinguishable inter-hemispheric structural connectivity between the patient and the healthy control group argues against the inter-hemispheric disconnection hypothesis of OMA [11, 12]. In line with the reduced intra-hemispheric structural connectivity, functional MRI revealed reduced activity in our patient’s FEF bilaterally. Additional support for a widespread intra-hemispheric oculomotor network impairment came from the observation that self-paced and saccades to remembered (imagined) targets were impaired which involve projections to the SC and PEF. Our results point to a patho-mechanism of OMA as a bilateral but ipsi-hemispheric oculomotor network disorder critically involving the FEF, at least in this novel *NPTX1* mutation with considerable gene expression in the frontal cortex.

As this implication from a single case study remains speculative, we recommend to apply the same imaging paradigms in the following patient cohorts in the future: (i) subjects carrying the missense mutation in the NPTX1 gene *without* OMA, (ii) OMA patients of other origins [in whom this missense mutation in the NPTX1 gene is ruled out, e.g., patients with oculomotor apraxia type 1 or 2 (AOA1, AOA2)], and (iii) OMA patients with focal structural lesions (e.g., bilateral vascular lesions in FEF, [6]) to look for the integrity of interhemispheric FEF connectivity.

## Supplementary Information

Below is the link to the electronic supplementary material.

Supplementary file1 (DOCX 47 KB)

Supplementary file2 MNI coordinates of the peak neural activity in the frontal (FEF) and supplementary eye field of the healthy control subjects and the patient (whole brain analysis). (PDF 189 KB)

Supplementary file3 The video shows behavioral abnormalities of the horizontal oculomotor apraxia of the patient in the following sequence (as listed in the slides between the video sections; the verbal instructions are displayed as subtitles) from two different perspectives (one from the investigator, the other from a third person’s perspective): voluntary (self-paced) visually guided vertical (1) and horizontal (2) saccades during head-stationary conditions, (3) in the head-free condition: combined horizontal saccade-head movements, (4) in the head-stationary condition: during horizontal and vertical smooth pursuit, (5) eye movements during the vestibulo-ocular reflex and (6) during visually guided hand movements without saccades (excluding optic ataxia). Under head-fixed conditions, there was severe horizontal OMA (no horizontal saccades and horizontal smooth pursuit). Under head-free conditions, visually guided horizontal saccades could be performed to visual targets in conjunction with large horizontal head movements. Vestibular responses during passive head movements reveals normal vestibular responses. (MP4 6255 KB)

## Data Availability

The data that support the findings of this study are available from the corresponding author upon reasonable request.
