# Supplementary material for: NPTX1-related oculomotor apraxia: an intra-hemispheric disconnection disorder
Source: J Neurol. 2022 Mar 14;269(7):3931–6. doi: 10.1007/s00415-022-11057-3 (PMC9217871; doi:10.1007/s00415-022-11057-3)
Supplement: Supplementary file 1 — Supplementary file1 (DOCX 47 KB) [file 415_2022_11057_MOESM1_ESM.docx]

**eMETHODS**

Structural [1] and functional [2] MR imaging was performed using a 3-T Siemens Magnetom Skyra scanner (Siemens, Erlangen, Germany) equipped with a 64-channel head-coil.

**Magnetic resonance imaging data acquisition**

Diffusion weighted images were acquired using pulsed gradient spin echo (PGSE) technique (TR = 5000 ms; TE = 77 ms; slices = 84; field of view = 234 mm; voxel resolution = 2 × 2 × 2 mm; readout bandwidth = 1630 Hz/pixels; 64-channel head coil; SENSE acceleration factor =3). Seven T2-weighted images without diffusion weighting (b = 0 s/mm^2^) were acquired, including one in opposite phase encoded direction. Furthermore, 101 images with noncollinear diffusion gradient directions distributed equally over the half-sphere covering 5 diffusion gradient strengths were obtained (b-values = [300.0, 700.0, 1000.0, 2000.0, 3000.0] s/mm^2^; shell-samples = [3, 7, 16, 29, 46]).

Structural images of the whole brain using a 3-dimentional (3D) T1-weighted MP-RAGE sequence were acquired (TR=1900 ms; TE=2.44 ms; TI=900 ms; flip angle 9°; 1×1×1 mm³ resolution; 192×256×256 mm³ field of view; acquisition time 6.08 minutes). Two runs of functional image recordings were acquired each applying a single-shot gradient-recalled echo-planar imaging (GRE-EPI) sequence sensitive to blood oxygen level dependent (BOLD) contrast (298 volumes, TR=1620 ms; TE=25 ms; flip angle=70°; in-plane resolution 2.5×2.5 mm²; 192×192 mm² field of view; 58 axial slices; 2.5 mm slice thickness, no interslice gap; simultaneous multi-slice factor 2; acquisition time: 8.05 minutes). Lights were switched off during recordings. Subjects were asked to keep their eyes open and to foveate a small red dot on a black background.

**Cortical and subcortical regions of interest (ROIs)**

The Destrieux atlas [3] was chosen to perform cortical parcellation using FreeSurfer (<https://surfer.nmr.mgh.harvard.edu>). Based on the *a priori* hypothesis, analysis of the functional MR imaging was performed in the frontal eye field (FEF) mask. Interhemispheric FEF-connectivity and the intrahemispheric connectivity of FEF with ipsilateral cortical and subcortical areas involved in the generation and control of eye movements were analyzed. This oculomotor network comprised the supplementary eye field (SEF), the parietal eye field (PEF), the dorsolateral prefrontal cortex (DLPFC), the caudate nucleus, the colliculus superior (SC) and the pons. For the FEF, SEF and PEF the masks of the precentral gyrus and sulcus, the frontal superior gyrus and the intraparietal sulcus were taken respectively. MNI coordinates of the peak activations were taken from fMRI studies reported in the literature [4-8] and the average was built. For the creation of the final regions of interest (FEF mask), we combined the obtained averaged activations from the FEF-related studies and the group average activation of our healthy age-matched control subjects during visually-guided saccades within the fMRI protocol by selecting the closest 700 voxels to those coordinates within the respective FreeSurfer mask. This resulted in the following MNI coordinates for FEF on both sides:-47 -6 54 for the left, and 44 -10 -61 for the right FEF. For the DLPFC, masks for the middle frontal gyrus as well as for the pons and the caudate nucleus were taken from FreeSurfer; the mask for the colliculus superior was drawn manually by one investigator (PJK). For the analysis of diffusion weighted imaging and structural connectivity individual masks were taken. Masks were coregistered to the diffusion space using fsl flirt/fnirt.[9] For the fMRI analysis, all individual FEF masks were coregistered to the MNI space in order to create one averaged FEF mask per hemisphere.

**Functional MRI analysis**

For the fMRI analysis, a first level analysis was applied contrasting visually guided, self-paced and imagined saccade to fixation periods. For the control group a second level group analysis was performed using a one-sample t-test. The group comparison (patient vs. healthy controls) in the whole brain analysis using SPM 12 revealed no significant voxels (FDR corrected statistics), likely to be due to the small sample size. Differences in saccade-related FEF / SEF activity are shown in Fig. 2 A and B (FDR corrected at p < 0.002, cluster size 20 voxel). Accordingly, contrast values were extracted from the FEF and SEF using the masks reported above (Fig. 2C) using MarsBar[10] within SPM. Results for FEF are shown in Fig. 2 D (similar results for SEF, not shown).

**Diffusion weighted imaging (DWI) analysis**

Preprocessing was performed using TOPUP and eddy.[11, 12] This includes motion artefact reduction, correction for field inhomogeneity, susceptibility-induced off-resonance field and eddy currents.

Multi-shell multi-tissue constrained spherical deconvolution [13, 14] was used to estimate the fiber orientation distributions within each voxel. Tissue partial volume estimates were obtained from FSL Fast. Whole-brain probabilistic tractography was performed using the second-order integration over fiber orientation distribution (iFOD2 [15]), initiating 10 million streamlines (i) in all voxels of the white matter as well as (ii) in voxels defined within the FEF-mask (s. above). Of the combined dataset, 5 million random streamlines were selected with both endpoints in the individual cortical or subcortical mask. Every streamline was weighted fitting the underlying diffusion compartment model using a Stick-Ball-Zeppelin model using COMMIT.[16] The stick compartment models the intra-axonal water with parallel diffusivity of 1.7E-3 mm^2^/s and no perpendicular diffusivity. The Ball compartment models the extra-axonal water with isotropic diffusivity of 1.7E-3 mm^2^/s and free water with diffusivity of 3.0E-3 mm^2^/s. The Zeppelin compartment adds an additional model of the extra-axonal water with parallel diffusivity of 1.7E-3 mm^2^/s and perpendicular diffusivity of 0.51E-3 mm^2^/s.

Streamlines connecting each pair of the defined FEF-mask were generated using the white matter query language [17] with the connected ROIs defined as endpoints. The summed COMMIT weight was generated for each connection to define the structural connectivity of that pair. To define intra- und interhemispheric connectivity, all COMMIT weights of connections with FEF were summed up.

**References**

1. Koch PJ, Park CH, Girard G*, et al* (2021) The structural connectome and motor recovery after stroke: predicting natural recovery. *Brain* 144: 2107-2119.

2. Helmchen C, Machner B, Rother M, Spliethoff P, Gottlich M, Sprenger A (2020) Effects of galvanic vestibular stimulation on resting state brain activity in patients with bilateral vestibulopathy. *Hum Brain Mapp* 41: 2527-2547.

3. Destrieux C, Fischl B, Dale A, Halgren E (2010) Automatic parcellation of human cortical gyri and sulci using standard anatomical nomenclature. *Neuroimage* 53**:** 1-15.

4. Anderson JC, Kennedy H, Martin KA (2011) Pathways of attention: synaptic relationships of frontal eye field to V4, lateral intraparietal cortex, and area 46 in macaque monkey. *J Neurosci* 31: 10872-10881.

5. Neggers SF, Diepen RM, Zandbelt BB, Vink M, Mandl RC, Gutteling TP (2012) A functional and structural investigation of the human fronto-basal volitional saccade network. *PLoS ONE*. 7: e29517.

6. Schraa-Tam CK, van der Lugt A, Smits M, Frens MA, van Broekhoven PC, van der Geest JN (2021) fMRI of optokinetic eye movements with and without a contribution of smooth pursuit. *J Neuroimaging* 18: 158-167.

7. Ohlendorf S, Sprenger A, Speck O, Glauche V, Haller S, Kimmig H (2010) Visual motion, eye motion, and relative motion: A parametric fMRI study of functional specializations of smooth pursuit eye movement network areas. *J Vis* 10: 21.

8. Thakkar KN, van den Heiligenberg FM, Kahn RS, Neggers SF (2014) Frontal-subcortical circuits involved in reactive control and monitoring of gaze. *J Neurosci*. 34: 8918-8929.

9. Jenkinson M, Bannister P, Brady M, Smith S (2002) Improved optimization for the robust and accurate linear registration and motion correction of brain images. *Neuroimage* 17: 825-841.

10. Brett M, Anton J-L, Valabregue R, Poline J-B (2002) Region of interest analysis using an SPM toolbox *Neuroimage* 16.

11. Andersson JLR, Sotiropoulos SN (2016) An integrated approach to correction for off-resonance effects and subject movement in diffusion MR imaging. *Neuroimage* 125: 1063-1078.

12. Smith RW, Yang BJ, Huang WD (2004). The measurement of solute diffusion coefficients in dilute liquid alloys: the influence of unit gravity and g-jitter on buoyancy convection. *Ann N Y Acad Sci* 1027: 110-128.

13. Jeurissen B, Tournier JD, Dhollander T, Connelly A, Sijbers J (2014). Multi-tissue constrained spherical deconvolution for improved analysis of multi-shell diffusion MRI data. *Neuroimage* 103: 411-426.

14. Tournier JD, Smith R, Raffelt D*, et al* (2019) MRtrix3: A fast, flexible and open software framework for medical image processing and visualisation. *Neuroimage*. 202: 116137.

15. Tournier JD (2019) Diffusion MRI in the brain - Theory and concepts. *Prog Nucl Magn Reson Spectrosc*. 112-113: 1-16.

16. Daducci A, Dal Palu A, Lemkaddem A, Thiran JP (2015) COMMIT: Convex optimization modeling for microstructure informed tractography. *IEEE Trans Med Imaging* 34: 246-257.

17. Wassermann D, Makris N, Rathi Y*, et al*  (2016) The white matter query language: a novel approach for describing human white matter anatomy. *Brain Struct Funct*. 2016 221: 4705-4721.
