# Supplementary material for: NPTX1-related oculomotor apraxia: an intra-hemispheric disconnection disorder
Source: J Neurol. 2022 Mar 14;269(7):3931–6. doi: 10.1007/s00415-022-11057-3 (PMC9217871; doi:10.1007/s00415-022-11057-3)
Supplement: Supplementary file 2 — Supplementary file2 MNI coordinates of the peak neural activity in the frontal (FEF) and supplementary eye field of the healthy control subjects and the patient (whole brain analysis). (PDF 189 KB) [file 415_2022_11057_MOESM2_ESM.pdf]

	region	X	Y	Z	T-values	Voxel	p(FDR) / p (uncorr) Cluster level
healthy controls	FEF (left)	-24	5	59	T = 7.75	0.04/0.002	33
healthy controls	FEF (right)	33	5	56	T = 7.16	0.10/0.011	21
healthy controls	SEF	3	-1	56	T = 11.91	0.001/<0.001	91
patient	FEF (left)	-51	-4	53	T = 9.67	<0.001/<0.001	45
patient	FEF (right)	51	2	41	T = 12.64	<0.001/<0.001	372
patient	SEF	-	-	-	-	-	-
